# Nuclear and Morphological Alterations in Erythrocytes, Antioxidant Enzymes, and Genetic Disparities Induced by Brackish Water in Mrigal Carp (*Cirrhinus mrigala*)

**DOI:** 10.1155/2022/4972622

**Published:** 2022-10-11

**Authors:** Ghulam Ali Raza, Abdul Ghaffar, Riaz Hussain, Adil Jamal, Zulfiqar Ahmad, Bahaeldeen Babiker Mohamed, Abdullah S. M. Aljohani

**Affiliations:** ^1^Department of Zoology, Islamia University of Bahawalpur, 63100, Pakistan; ^2^Department of Pathology, Faculty of Veterinary Sciences, Islamia University of Bahawalpur, 63100, Pakistan; ^3^Sciences and Research, College of Nursing, Umm Al Qura University, 715 Makkah, Saudi Arabia; ^4^Department of Food Science and Technology, Faculty of Agriculture and Environment, The Islamia University of Bahawalpur, 63100, Pakistan; ^5^Institute of Environment Natural Resources, The National Centre of Research, Khartoum, Sudan; ^6^Department of Veterinary Medicine, College of Agriculture and Veterinary Medicine, Qassim University, Buraydah, Saudi Arabia

## Abstract

Salinization of aquatic ecosystem, abrupt climate change, and anthropogenic activities cause adverse impact on agricultural land/soil as well as the aquaculture industry. This experimental study was designed to evaluate different biomarkers of oxidative stress, antioxidant enzymes, and genotoxic potential of diverse salinities of brackish water on freshwater fish. A total of 84 fresh water mrigal carp (*Cirrhinus mrigala*) were randomly segregated and maintained in four groups (T0, T1, T2, and T3) in a glass aquarium under similar laboratory conditions at various salinity levels (0, 3, 5, and 7 parts per thousand) to determine the pathological influence of brackish water. All the fish in groups T1, T2, and T3 were exposed to various salinity levels of brackish water for a period of 90 days while the fish of group T0 served as the control group. The experimental fish reared in different groups T1, T2, and T3 displayed various physical and behavioral ailments. The results revealed significantly augmented quantity of different oxidative stress indicators including reactive oxygen species (ROS) and thiobarbituric acid reactive substance (TBARS) in different visceral tissues (kidneys, liver, and gills) of exposed fish. Different antioxidant enzymes such as reduced glutathione (GSH), peroxidase (POD), superoxide dismutase (SOD), and catalase (CAT) along with total proteins were remarkably reduced in the kidneys, gills, and liver tissues. Results showed significantly increased values of different nuclear abnormalities (erythrocyte with micronucleus, erythrocyte with condensed nucleus, and erythrocyte with lobed nucleus) and morphological changes (pear shaped erythrocyte, spindle-shaped erythrocytes, and spherocyte) in red blood cells of experimental fish. The results on genotoxic effects exhibited significantly increased DNA damage in isolated cells of liver, kidneys, and gills of exposed fish. The findings of our experimental research suggested that brackish water causes adverse toxicological impacts on different visceral tissues of fresh water fish at higher salinity level through disruption and disorder of physiological and biochemical markers.

## 1. Introduction

Aquaculture is considered the most important source of proteins for human as well as other terrestrial animals. The sustainable production and utilization of nutritive foods has become crucial due to rapid growth of human population. Fish and different other aquaculture species play important role to control malnutrition across the globe. Due to extensive growth of human population, if food supply and security cannot be maintained, it may lead to numerous problems regarding utilization of nutritive foods. Therefore, optimum production and health of aquaculture has become necessary due to the increase pressure of human population in various developing countries [1]. Studies have investigated that global aquaculture supplies huge amount of proteins in terms of growing different kinds of fish in a very short period by providing a healthy and perfect habitat [2–4].

An ever-increasing human population, water scarcity, land acquisition costs, and the indiscriminate use of pharmaceuticals and variety of chemicals are putting pressure on the aquaculture industry [5]. Physiological functions, growth performance, and immunological modulation in fish farming are the key factors influenced by water having high salinity [6]. Saltiness is one of the most important feature characteristics in determining osmoregulatory stress level in different species of freshwater fish. It is observed that investigation of adverse effects of water having high salinity is important to maintain the process of osmoregulation and ionic concentration in various tissues (gill, kidney, and gut) of fish for optimum biological functions and growth [7].

When compared to freshwater, brackish water has high salinity levels but is less salty than seawater [8]. There are a variety of sources of brackish water, including groundwater (naturally salinized aquifers), rivers, wastewater (including those produced by hydraulic fracturing, human activities, and industrial operations) and irrigation return flow [9]. The runoff of various salt deposits such as halite or gypsum is found in sedimentary rocks, as well as discharges from industrial effluents containing dissolved salts and leachates from saline soils ultimately lead to formation of brackish water [10, 11]. Salinity is believed to have an impact on around one-third of the world's cultivable land [12].

Understanding of optimum salinity for specific species of fish and other species can contribute in expansion of aquaculture productions by utilizing wide range of culturing environments including resources of brackish water. Rearing of both freshwater and marine fish species in water having inadequate salinity levels can directly affects the physiology of fish causing poor growth, increased mortality, low immune response, and disease resistance [13].

However, prior to establishing aquaculture operations in these places, it is critical to pick suitable fish species that are tolerant to saline environments. This is mostly due to the fact that greater salinity of water can operate as a significant stressor in both wild and farmed environments [14]. Furthermore, salinity has the ability to influence the metabolism of fish, which in turn has an impact on the survival, growth, feed intake, and even the geographic spread of fish species [15, 16].

Blood is commonly regarded as an effective stress indicator for revealing the potential harmful stress and health status of fish [17–19]. It is recorded that the monitoring and determination of hematological profile of fish is of vital, useful, and reliable tool for the estimation of different pathological ailments induced by various environmental pollutants both in aquatic and terrestrial animals. In addition, studies have highlighted that determination of variations in different biochemical profile including enzymes and proteins is of vital importance to assess the physiological ailments in aquatic organisms [17, 20–23]. The red blood cells of fish and other animals are directly exposed to different environmental contaminants when they enter into the blood and are reliable biomarkers for the early screening of pathophysiological effects employed by all kind of drugs and other pollutants [18, 20–23]. The toxic effects induced by various pollutants can induce physiological stress leading to induction of different genotoxic abnormalities and development of morphological nuclear changes in erythrocytes of exposed aquatic animals [17, 24]. Studies have recorded that investigation of morphological features of red blood cells are frequently utilized in toxicity testing and in determination of health condition of fish [17, 18].

Reactive oxygen species (ROS) generate oxidative stress when they be more than the body's antioxidant capacity, as they do under conditions of environmental stress [25]. ROS generation is tightly linked to variations in salinity, resulting in oxidative stress in fish [26, 27]. It has been recorded that ROS are extensively generated in the body during an aerobic condition and at different physiological disorders creating oxidative eustress [17, 21, 22]. Moreover, different antioxidant enzymes including both enzymatic and nonenzymatic play crucial and vital role in maintaining the redox status and oxidative stress balance by providing defense against free radicals [17, 22]. Different antioxidant enzymes (SOD, CAT, and GSH) prevent induction of oxidative stress in different tissues of the animals including aquatic life and are frequently used to estimate the risk of various environmental contaminants in [17, 21]. It has been recorded that increased salinity level induces different physiological disorders via oxidative stress and alterations in concentrations of hormones in plasma, abnormal energy metabolism, and electrolyte imbalance in different animal species [28]. In addition, different investigations have indicated that increased level of salinity results disorders regarding intake of O_2_ leading to abnormalities in respiration which can be transformed to oxidative stress and lower the antioxidant defense [29].

As a result, it is vital to investigate the specific stresses associated with brackish water salinity. So, the objective of this study is to examine the erythrocytic morphological and nuclear alterations, genotoxic potential, oxidative stress, and status of antioxidant enzymes in visceral tissues of mrigal carp reared in brackish water.

## 2. Methodology

### 2.1. Fish Handling

The recent research was conducted in laboratories of the Department of Zoology and Department of Pathology, Islamia University of Bahawalpur. A total of 84 mrigal fish with approximately similar body weight (160-180 g), size, and free from any external and internal parasitic infections were obtained from private hatchery located at district Bahawalpur. All the Fish were carried to laboratory in oxygen rich plastic sacks and were kept in glass aquaria under similar laboratory conditions. Aerators were installed in all the glass aquaria to ensure availability of sufficient oxygen for experimental organisms. After a period of 15 days of acclimatization, all the fish were indiscriminately picked and placed in four groups. A total of 21 fish were placed in each group. All of the fish were fed standard fish feed with enough proteins (25 percent). All experimental procedures were according to the rules issued by the office of the directorate of research and bioethics committee of Islamia University Bahawalpur concerning the use and welfare of laboratory organisms. On a daily basis, all waste material (fish excretions) and residual feed were filtered and removed from each aquarium. Different physical and chemical properties of study water were determined on weekly basis, and the average values were computed at days 30, 60, and 90 of the trial. All the chemicals used in our trial were obtained from Sigma-Aldrich (USA) and Merck (Germany).

### 2.2. Collection of Water

**Figure 1:**
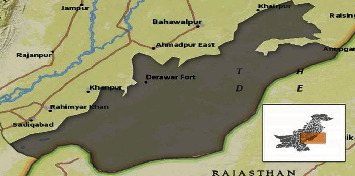
Geographical location of brackish water of selected site (Derawer Fort) of District Bahawalpur, Punjab, Pakistan.

The brackish water used in present research was collected from Derawer Fort (Cholistan desert) District Bahawalpur, Punjab, Pakistan. Derawer fort is a well-known historical place being in Cholistan desert and lies between latitudes 28.76° N and longitudes 71.33° E (Figure 1). Brackish water having salinity level of 7.2 parts per thousand (ppt) was collected and diluted to 3.00 ppt, 5.00 ppt, and 7.00 ppt by adding fresh water (Table 1), whereas fresh water (0 ppt) was obtained from the laboratory, Department of Zoology, Islamia University of Bahawalpur.

### 2.3. Experimental Design

After two weeks of acclimatization, all the active fish, without any obvious clinical ailments and free from any external and internal parasitic infection, were blindly picked and placed into four equal groups (T0, T1, T2, and T3). Fish kept in groups (T1, T2, and T3) were exposed to brackish water having different salinity levels (3.00 ppt, 5.00 ppt, and 7.00 ppt). The fish present in group T0 served as control group (0 ppt). All of the fish were closely observed on a regular basis by visual examination for any obvious physical or behavioral alterations.

### 2.4. Blood Sampling and Genotoxicity Evaluation

By using 26 gauge sterile hypodermic needle, blood sample (2 mL) was obtained from the caudal vein of each fish at days 30, 60, and 90 of the trial [30]. After that, blood films were made on glass slides from fresh blood without any anticoagulant. All the blood films were then dried, fixed with 100% alcohol, and stained using Giemsa solution. All the blood films were observed under computer-assisted light microscope to record the nuclear and morphological disparities in erythrocytes. A total of 1500 erythrocytes from each fish were observed with the help of computer-assisted light microscope using immersion lens [30]. The severity of genotoxic effects of study water in visceral organs (kidneys, liver, and gills) was determined by using single-cell gel electrophoresis or comet essay [31, 32]. For this purpose, visceral organs (kidneys, liver, and gills) were removed after dissection and placed in chilled normal saline solution. After that, about 0.2 g tissue from each organ (kidneys, liver, and gills) was homogenized and centrifuged separately for separation of cells. DNA damage in isolated cells was determined by using a comet assay [31]. Briefly, thin smear of low melting point (1%) along with normal point (1%) agarose liquified in Milli-Q water was created on frosted slides. Isolated cells of each tissue were suspended in agarose of low melting point [31]. After preparing the slides, they were lysed in cold buffer solution. For electrophoresis, the slides were placed in a horizontal tank with a chilled electrophoretic solution. The electrophoresis (25 volts) was conducted for a period of 30 minutes [33]. After the electrophoresis, the prepared slides were neutralized by placing in an ice-cold 0.4 M Tris buffer (pH 7.5) [34]. Eventually, ethidium bromide was applied for staining, and frequency of DNA damage in different tissues was estimated by using a fluorescent microscope (Euromex Ox Spare LED; SL.5510) at 400x. A total of 500 cells/fish/slides were examined, and frequency of DNA damage (%) was calculated [34].

### 2.5. Tissue Preparation and Biochemical Analysis

Fish was dissected at days 30, 60, and 90 of the experiment for separation of different visceral organs (kidneys, gills, and liver) for biochemical evaluation. All the collected tissues were placed in cold saline solution for estimation of thiobarbituric acid reactive substance (TBARS), reactive oxygen species (ROS), reduced glutathione (GSH), total proteins, and various antioxidant enzymes catalase (CAT), peroxidase (POD), and superoxide dismutase (SOD) [34]. Various biochemical markers, such as reactive oxygen species [35] and thiobarbituric reactive substance [36], along with reduced glutathione [37], were estimated in the kidneys, gills, and liver of each fish using UV spectrophotometer (Libra S60; Biochrom, UK). The status of antioxidant enzymes including peroxidase [38], superoxide dismutase [39], and catalase [38] in the liver, gills, and kidneys was determined following the procedures as described in the previous published literature.

### 2.6. Statistical Analysis

All the collected data is presented as a mean standard deviation. Variance of one-way analysis was used in IBM SPSS statistical programmer (version no. 20) to conduct statistical analysis because the data in each group was normally distributed. A post hoc Tukey's test with significance threshold of 0.05 was used to evaluate the variance in mean values (mean ± SE) of erythrocytic nuclear and morphological disparities, oxidative stress, and antioxidant enzymes, along with genotoxic potential in visceral organs of control and experimental groups. Pearson's correlation analysis was performed to know the association between different variables of the liver, kidneys, and gills of experimental fish.

## 3. Results

No mortalities were recorded in fish kept in various treatment groups (T1, T2, and T3) having different salinity levels of brackish water throughout the study. Mild to moderate abnormal swimming, lack of coordination, air gulping, swimming in isolation, and lie down on single side were visually observed in experimental fish exposed to brackish water of high salinity (T2) after day 60 of the trial. Different severe physical disorders such as increased swimming, protrusion of eyes, loss of stability, increased opercular movements, breathing on surface of water, and mucous discharge from mouth and gills were observed in fish of group T3 after day 60 of experiment. The intensity of several behavioral and clinical indications in fish exposed to brackish water increased in a time-dependent manner.

### 3.1. Morphological and Nuclear Irregularities in Erythrocytes

**Figure 2:**
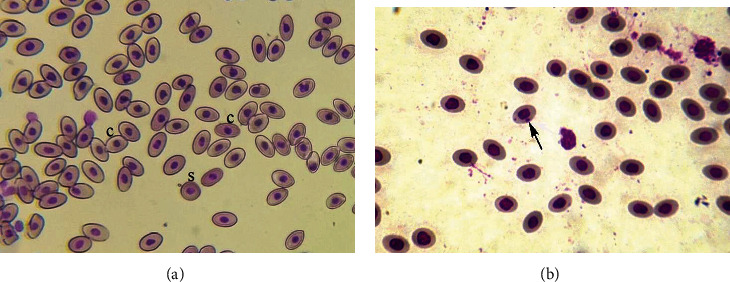
Photomicrograph of blood smear of fish placed in group T3 at day 90 of trial showing (a) erythrocyte with condensed nucleus (c) and spherocyte (s) and (b) showing erythrocyte with micronucleus (arrows). Giemsa stain: ×1000.

The results of the current study revealed that the frequency of various nuclear and morphological abnormalities in erythrocytes of fish exposed to increasing salinity levels of brackish water was remarkably increased in comparison to normal fish. The prevalence of different morphological anomalies in erythrocytes was significantly high in fish exposed to 5.00 ppt and 7.00 ppt salinity of brackish water at days 60 and 90 of current study. The percentage of erythrocytes with lobed nuclei, erythrocytes with blebbed nuclei, erythrocytes with vacuolated nuclei, erythrocytes with notched nuclei, and erythrocytes with binucleus was considerably high in mrigal carp exposed to 5.00 ppt and 7.00 ppt salinity at days 60 and 90 of the experiment comparing to control group (Table 2). On days 60 and 90 of the current study, fish exposed to 5.00 ppt and 7.00 ppt salinity of brackish water had also significantly higher percentage of erythrocytes with micronucleus (Figure 1) along with erythrocytes with condensed nucleus than untreated control fish (Table 2). Increased percentile rate of erythrocytes having spindle shape and spherocytes (Figure 2) was recorded in mrigal carp exposed to 5.00 ppt and 7.00 ppt salinity of brackish water at days 60 and 90 of the trial (Table 2).

### 3.2. Oxidative Stress and Status of Antioxidant Enzymes

The current study revealed that the quantity of ROS in isolated hepatocytes of fish exposed to 5.00 ppt and 7.00 ppt salinity of brackish water increased significantly at days 60 and 90, while the contents of TBARS in isolated hepatocytes of mrigal carp treated with 7.00 ppt salinity of brackish water was significantly higher at days 60 and 90 of trial. The contents of TBARS in isolated hepatocytes of mrigal carp treated with 5.00 ppt was considerably high at day 90 of the trial (Table 3). The contents of total proteins along with reduced glutathione in isolated hepatocytes of mrigal carp reared in brackish water having 7.00 ppt salinity was prominently diminished at days 60 and 90 of the trial (Table 3), while quantity of reduced glutathione and total proteins in isolated hepatocytes of mrigal carp in 5.00 ppt salinity was significantly decreased at day 90 of the trial (Table 3). When compared to untreated mrigal carp, the quantity of SOD, CAT, and POD in isolated hepatocytes of treated fish exposed to 5.00 ppt and 7.00 ppt salinity of brackish water was significantly lowered at days 60 and 90 of the trial (Table 3).

At days 60 and 90 of the trial, the contents of ROS in isolated kidneys of fish exposed to 7.00 ppt salinity of brackish water was considerably increased, while the contents of ROS in isolated kidney cells of fish exposed to 5.00 ppt was significantly increased at day 90 of the experiment (Table 4), while the contents of TBARS in isolated kidneys of fish exposed to 7.00 ppt salinity was significantly increased at days 60 and 90 of the current study (Table 4). The contents of total proteins and reduced glutathione were decreased considerably in isolated kidney of fish exposed to 5.00 ppt and 7.00 ppt salinity of brackish water at day 90 of the trial (Table 4). The values of SOD were decreased in isolated kidneys of fish exposed to 7.00 ppt salinity at days 60 and 90 of the trial, while the values of SOD decreased at 5.00 ppt salinity at day 90 of the trail (Table 4). At day 90 of the trail, the contents of CAT and POD decreased in isolated kidney of fish exposed to 5.00 ppt and 77.00 ppt salinity of brackish water (Table 4).

The values of ROS in isolated gill tissues of fish exposed to 5.00 ppt and 7.00 ppt were significantly increased at days 60 and 90 of the trial, while contents of TBARS gill of fish were increased significantly when exposed to 5.00 ppt salinity at 90 days of the trail and 7.00 ppt salinity at days 60 and 90 of the trial (Table 5). The contents of GSH were decreased in isolated gills of fish exposed to 5.00 ppt and 7.00 ppt salinity of brackish water at days 60 and 90 of the current study (Table 5). At days 60 and 90 of the trial, total proteins in isolated gill of fish exposed to 7.00 ppt were significantly decreased (Table 5). The contents of total proteins decreased considerably in the gills of mrigal carp reared in brackish water having 5.00 ppt salinity at day 90 of the experiment. The concentrations of antioxidant enzyme like SOD, CAT, and POD in isolated gills decreased significantly at days 60 and 90 of the trail in fish exposed to 5.00 ppt and 7.00 ppt salinity of brackish water (Table 5).

### 3.3. Genotoxic Potential

**Figure 3:**
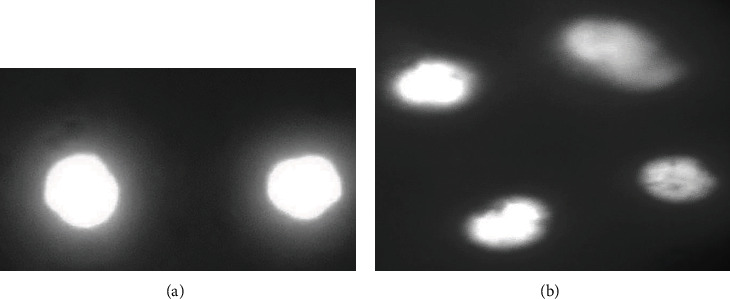
Comet assay or single cell gel electrophoresis exhibiting normal cells (a) and DNA damage in isolated cells of fish kept in group T3 at day 90 of trial. Ethidium bromide stain: 400x.

**Figure 4:**
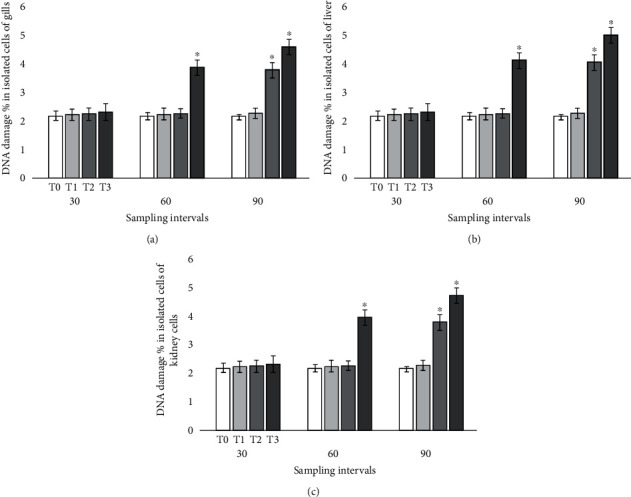
Photograph exhibiting frequency of DNA damage in isolated cells of gill (a), liver (b), and kidneys (c) of fish reared in brackish water. Bars show mean ± SE, and asterisks on bars show significant difference at (*p* < 0.05).

Results on the DNA damage determined by comet assay (Figure 3) showed significantly increased frequency of DNA damage in various visceral organs of treated fish at days 60 and 90 in isolated cells of the hepatocytes, gills, and kidneys. At days 60 and 90 of the experiment, the frequency of genotoxic potential in isolated gill cells of mrigal carp exposed to 7.00 ppt salinity increased dramatically. The results recorded that the DNA damage was increased significantly at day 90 of the experiment in mrigal carp exposed to 5.00 ppt salinity of brackish water (Figure 4). The frequency of mutagenic potential in isolated hepatocytes and kidney cells at days 60 and 90 in fish exposed to brackish water having 7.00 ppt salinity was significantly high than the fish of control group. The frequency of DNA damage was also higher in fish exposed to brackish water having 5.00 ppt at day 90 of the experiment.

### 3.4. Correlation Analysis of Studied Variables

**Figure 5:**
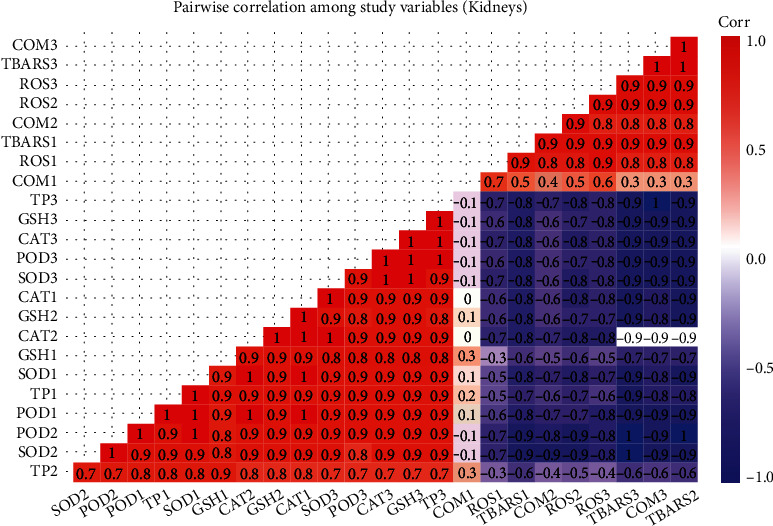
Photograph shows correlation between study variables of kidneys at different levels of sampling days. The values of correlation ranged from -1 to **+**1. The absolute values between 0 and 0.2, 0.2 and 0.4, 0.4 and 0.6, 0.6 and 0.8, and 0.8 and 1 indicate very week, week, moderate, strong, and very strong relationship, respectively. The last digit of each variable indicates sampling points which are 1 (day 30), 2 (day 60), and 3 (day 90).

**Figure 6:**
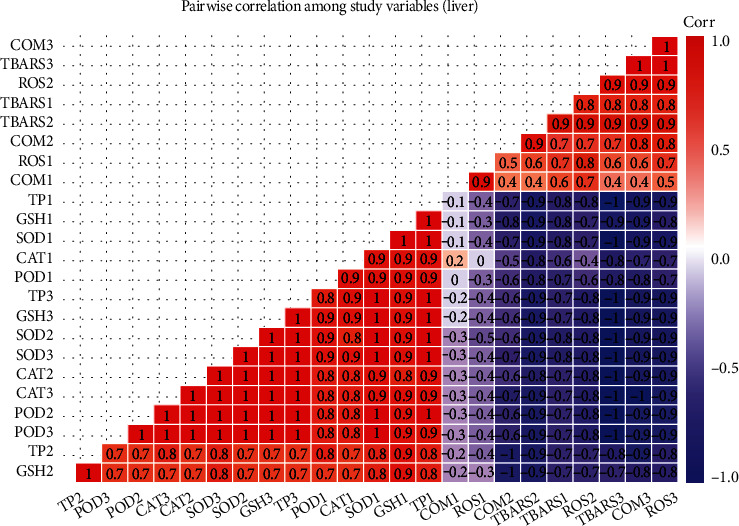
Photograph shows correlation between study variables of liver at different levels of sampling days. The values of correlation ranged from -1 to **+**1. The absolute values between 0 and 0.2, 0.2 and 0.4, 0.4 and 0.6, 0.6 and 0.8, and 0.8 and 1 indicate very week, week, moderate, strong, and very strong relationship, respectively. The last digit of each variable indicates sampling points which are 1 (day 30), 2 (day 60), and 3 (day 90).

**Figure 7:**
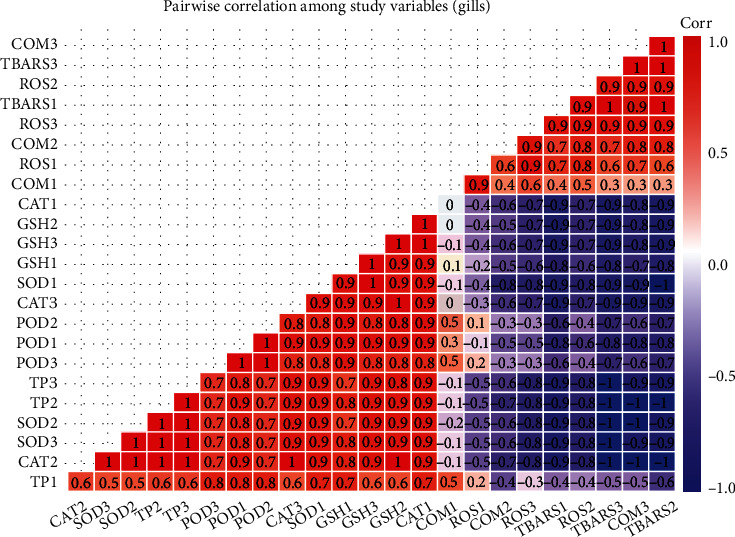
Photograph shows correlation between study variables of gills at different levels of sampling days. The values of correlation ranged from -1 to **+**1. The absolute values between 0 and 0.2, 0.2 and 0.4, 0.4 and 0.6, 0.6 and 0.8, and 0.8 and 1 indicate very week, week, moderate, strong, and very strong relationship, respectively. The last digit of each variable indicates sampling points which are 1 (day 30), 2 (day 60), and 3 (day 90).

The results on Pearson's correlation analysis showed correlation between study variables of the kidneys, liver, and gills of fish reared in brackish water at different sampling days. The results exhibited that total proteins in the kidneys of fish at day 60 has correlation of 0.7 with superoxide dismutase. Weak correlation was observed between DNA damage (COM) by comet assay with other variables. Reactive oxygen species and TBARS at sampling day 30, DNA damage by comet assay, TBARS, and ROS at day 60, while ROS, TBARS, and DNA damage by comet assay at day 90 have significant negative correlation. The variables POD, TP, SOD, GSH, and CAT at day 30, variables SOD, POD, CAT, and GSH at day 60, and variables SOD, POD, CAT, GSH, and TP at day 90 of trial have significant positive correlation (Figure 5). The results on Pearson's correlation analysis for different variables of liver showed weak correlation between frequency of DNA damage at sampling day 30 and all other variables. The results indicated that total proteins (TP), POD, CAT, SOD, and GSH at sampling day 30, TP, CAT, POD, and SOD at sampling day 60, and POD, CAT, SOD, GSH, and TP have significant positive correlation. Results indicated that ROS and TBARS at day 30, percentile rate of DNA damage, TBARS, and ROS at day 60, and TBARS, percentile rate of DNA damage, and ROS at sampling day 90 have negative correlation (Figure 6). The results on Pearson's correlation analysis for different variables of gills showed weak correlation between frequency of DNA damage at sampling day 30 and all other variables. The results indicated that different variables such as CAT, SOD, GSH, and POD at day 30, GSH, CAT, SOD, and TP at day 60, and SOD, TP, POD, POD, CAT, and GSH at day 90 of trial have significant positive correlation. Results showed that variable including ROS and TBARS at day 30, percentile rate of DNA damage (COM), ROS and TBARS at day 60, and ROS, TBARS, and COM at day 90 of trial have negative correlation (Figure 7).

## 4. Discussion

In brackish and marine water, salinity is an essential physical feature that is employed as a water tracer. It denotes the overall concentration of dissolved inorganic ions and salts. Salinization is a natural rise in soluble salts over an optimal level. Salinity is a vital environmental component for all aquatic organisms including fish. Abrupt change in the salinity of habitat water generates salinity stress by interfering with physiological homeostasis and normal biological activities of the exposed animals. The majority of wetlands is degraded due to salinization which ultimately leads to an imbalance in the ecosystem and impacts the biodiversity [40]. Salinization is principally caused by glacial/interglacial cycles, catchment weathering, salt content in rain (acquired by evaporation), and sea spray [40, 41]. Moreover, salinization occurs as a result of anthropogenic activity such as urban drainage inflow, vegetation clearing, intense irrigation, river management, sea level rise caused by global warming, and mining activities. It has been recorded that salinization results in biodiversity loss and environmental imbalance [42, 43].

After prolonged exposure to different salinities, mrigal carp displayed several behavioral abnormalities including abnormal swimming pattern, gill motion, and convulsions. The severity of these anomalies increased in fish reared in brackish water in a time- and concentration-dependent manner. Formerly, different physical anomalous-like abnormal swimming pattern, gill motion, and convulsions in *Pangasionodon hypophthalmus* [44] at higher salinities have been observed. Abnormal swimming pattern has been detected in Indian major carp, *Catla catla* [45], *Oryzias latipes* [46], and *Cyprinus carpio* [47]. Previously, disorders of convulsions were detected in *Channa punctatus* reared in water at high level of salinity [48].

Nuclear and morphological abnormalities in red blood cells of fish and other avian species are regarded as reliable biomarkers which provide an overview of genetic alterations and cytotoxicity induced by different natural and synthetic environmental pollutants [34]. It has been recorded that estimation of morphological and genetic disparities in erythrocytes are effective indicators of oxidative stress [49]. The current study recorded significantly higher frequency of different nuclear and morphological alterations including erythrocytes with lobed nuclei, erythrocytes with blebbed nuclei, erythrocytes with vacuolated nuclei, erythrocytes with notched nuclei, erythrocytes with micronuclei, erythrocytes with condensed nucleus, and binucleated erythrocytes along with different morphological changes such as pear-shaped erythrocytes and spherocytes in exposed fish. As far as we know, no information related to erythrocytic morphological and nuclear changes in mrigal carp reared in brackish water is available. According to previous studies, it has been recorded that erythrocytes are the best indicators of oxidative stress showing almost all morphological and nuclear alterations in animals exposed to various toxicants [50–52]. Increased intracellular generation of reactive oxygen and nitrogenous species might be the cause of these cellular alterations [53, 54]. In our study, increased percentage of erythrocyte with micronuclei and lobed nuclei might be owing to an excess of caspase-activated DNase, which is accountable for the cleavage of cytoskeletal (gelsolin, fodrin, and vimentin), nuclear, and aneuploid proteins, as well as oxidative damage to the mitochondrion [55, 56]. Furthermore, different studies have observed different morphological and nuclear alterations in erythrocytes of various species of fish (*Cyprinus carpio* and *Labeo rohita*) and other avian species in response to oxidative stress [30, 51, 52, 57–61]. The increase lipid peroxidation product causes increased permeability and decreased symmetry of the membrane of red blood cells resulting in increased osmotic fragility and abnormalities in the surface of erythrocytes [20, 23].

Antioxidant enzymes including GSH, SOD, CAT, and POD along with oxidative stress (TBARS and ROS) parameters are well-known indicators of inflammatory reactions and effective tools for monitoring of tissues from free radical injuries [32, 60, 62, 63]. These parameters are frequently measured to assess toxicological consequences of various hazardous environmental and synthetic compounds [64, 65]. Our study results exhibited significantly increased contents of oxidative stress biomarkers including reactive oxygen species (ROS) and thiobarbituric reactive substance in different visceral tissues like gills, liver, and kidneys of mrigal carp reared in brackish water. Published data has indicated that exposure to different toxicants induces rapid generation of reactive oxygen species in target and nontarget animals [34]. The generation of reactive oxygen species (ROS) initiates the lipid peroxidation process which ultimately leads to induction of abnormalities in cellular membranes of various cells of exposed tissues causing severe damage to these tissues and formation of thiobarbituric reactive substance [61, 66]. Previously, it has been recorded that salinity is the major threshold for increased production of ROS and TBARS in *Dicentrarchus labrax* [67], *Catla catla* [68], and Esturine fish [69]. In our study, elevated levels of oxidative stress parameters in mrigal carp reared in brackish water of various salinities might be related to the depletion and misbalancing of antioxidant enzymes. Furthermore, the elevated contents of oxidative stress parameters might be attributed to tissue damage and abnormal mechanism of oxidative phosphorylation [61]. Our research indicated that contents of GSH and total proteins were decreased in tissues including gills, liver, and kidneys of mrigal carp reared in brackish water. Previously, no information could be found regarding the lower contents of GSH and total proteins in various tissues of mrigal fish due to brackish water. The decreased levels of GSH and total proteins in current study might be due to dysfunction of tissues and increased consumption of energy to eliminate the oxidative stress. Several contaminants have previously been linked to protein deprivation in the liver, gills, and kidneys of numerous fish such as *Labeo rohita*, *Channa punctatus*, *Mystus vittatus*, and *Oreochromis spilurus* [66, 70, 71]. The contents of different antioxidant enzymes including reduced glutathione, superoxide dismutase, catalase, and peroxidase in different visceral tissues (gills, liver, and kidneys) were significantly lowered in mrigal fish reared in brackish water. Previously, it has been recorded that the contents of GSH, SOD, CAT, and POD were decreased in *Cyprinus carpio* reared in water of various salinities [72]. In previous published literature, it has been recorded that the increased contents of oxidative stress parameters and reduced contents of antioxidant enzymes in tissues of visceral organs of treated fish are interlinked [66, 72, 73]. Moreover, lower contents of antioxidant enzymes in tissues (liver, gills, and kidneys) of cat fish [74], reduced glutathione in the liver of Delta smelt [75], Jundiara fish [76], and lower values of reduced glutathione and superoxide dismutase in plasma of gold fish [77] due to various toxicants have been recorded. In contrast to our results, increased contents of superoxide dismutase, peroxidase, reduced glutathione, and catalase in different species of fish including Nile tilapia [78], *Catla catla* [68], *Anoplopoma fimbria* [27], and *Clarias gariepinus* [79] reared in water having higher salinity have been recorded.

In our research, the results on comet assay under alkaline conditions displayed high frequency of DNA damage in isolated cells of the kidneys, gills, and liver of Mrigal fish reared in brackish water. It has been determined that comet assay is a sensitive, appropriate, reliable, and frequently used approach for assessing DNA damage in various tissues of aquatic and terrestrial species [30, 32, 60, 66, 80]. Previously, no report is available in published literature regarding DNA damage in various tissues of freshwater fish reared in brackish water. Our study investigated significantly increased DNA damage in mrigal carp reared brackish water. The increased frequency of DNA damage in multiple tissues of Mrigal fish might be due to increase production of free radicals leading to oxidative stress. Furthermore, genotoxic effects can be primarily linked to oxidative stress [17, 62, 81] and lipid peroxidation which might be the source genotoxicity [20–23]. Various previous reports have indicated that the production of free radicals along with oxidative stress are the primary causes of genotoxicity in different tissues of animals [31, 32, 59, 60, 66]. Moreover, DNA damage in various visceral organs (liver, kidneys, and gills) of mrigal fish might be linked to genetic mutations generated by brackish water causing abnormal physiological disorders in different tissues and abnormal functions of various proteins resulting in dysfunctioning of mitochondrion and breakdown of nuclear proteins. The results of this experimental study suggested that brackish water causes adverse toxicological problems on blood and various visceral tissues of mrigal carp (*Cirrhinus mrigala*). Exposure of mrigal carp to brackish water at 5.00 ppt and 7.00 ppt induces genotoxic effects in isolated cells of the liver, kidneys, and gills. Furthermore, the results of our study indicated that brackish water even having low salinity levels reduces different antioxidant biomarkers and increases oxidative stress biomarkers in multiple visceral organs of fresh water fish mrigal carp when reared for long duration.

## Figures and Tables

**Table 1:** Physico-chemical properties of water in various salinity.

Parameters	Salinity levels			
	T0	T1	T2	T3
Electrical conductivity (mS/cm)	5.73	30.7	82.8	104.6
Water temperature (°C)	26.8	27.2	26.2	28.1
Total dissolved solids (mg/L)	172.7	195.7	212.7	228.4
Dissolved oxygen (mg/L)	6.97	7.11	7.33	7.38
pH	7.5	7.7	7.9	8.1
Total hardness (mg/L)	291.45	515.62	711.8	1036.4
Chlorides (mg/L)	48.6	472.3	758.5	1037.7
Magnesium (mg/L)	72.63	112.7	197.4	242.9
Calcium (mg/L)	44.56	59.5	77.3	104.8
Potassium (mg/L)	3.36	27.6	33.8	38.9
Sulphates (mg/L)	7.87	12.6	15.7	17.4
Sodium (mg/L)	43.86	211.5	387.3	667.9
Total alkalinity (CaCO_3_ mg/L)	273.89	321.5	335.8	360.3

T: treatments; T0: 0.00.00 ppt; T1: 3.00 ppt; T2: 5.00 ppt; T3: 7.00 ppt.

**Table 2:** Frequency of different morphological and nuclear changes in erythrocytes of mrigal carp (*Cirrhinus mrigala*) due to brackish water of different salinity regimes.

Parameters/days	Groups (brackish water of different salinities)			
	T0 (0.0 ppt)	T1 (3.0 ppt)	T2 (5.0 ppt)	T3 (7.0 ppt)
*Erythrocytes with lobed nucleus (%)*				
30	1.33 ± 0.17	1.39 ± 0.18	1.43 ± 0.18	1.46 ± 0.13
60	1.35 ± 0.09	1.41 ± 0.11	2.36 ± 0.22^∗^	2.92 ± 0.24^∗^
90	1.34 ± 0.13	1.44 ± 0.15	2.87 ± 0.21^∗^	3.64 ± 0.18^∗^
*Erythrocytes with blebbed nucleus (%)*				
30	1.25 ± 0.10	1.30 ± 0.22	1.31 ± 0.13	1.32 ± 0.17
60	1.25 ± 0.11	1.31 ± 0.21	2.20 ± 0.35^∗^	2.31 ± 0.29^∗^
90	1.26 ± 0.14	1.32 ± 0.44	2.44 ± 0.11^∗^	2.88 ± 0.58^∗^
*Erythrocytes with vacuolated nucleus (%)*				
30	2.35 ± 0.25	2.37 ± 0.18	2.41 ± 0.17	2.42 ± 0.25
60	2.36 ± 0.23	2.39 ± 0.18	3.11 ± 0.31^∗^	3.66 ± 0.30^∗^
90	2.39 ± 0.28	2.40 ± 0.39	3.43 ± 0.26^∗^	4.3 ± 0.25^∗^
*Erythrocytes with notched nucleus (%)*				
30	1.78 ± 0.15	1.81 ± 0.18	1.82 ± 0.83	1.84 ± 0.21
60	1.79 ± 0.17	1.83 ± 0.15	2.65 ± 0.34^∗^	3.14 ± 0.26^∗^
90	1.80 ± 0.18	1.85 ± 0.13	3.13 ± 0.17^∗^	3.82 ± 0.24^∗^
*Binucleated erythrocytes (%)*				
30	1.64 ± 0.32	1.66 ± 0.28	1.68 ± 0.35	1.72 ± 0.25
60	1.69 ± 0.32	1.68 ± 0.28	2.42 ± 0.27^∗^	2.93 ± 0.17^∗^
90	1.71 ± 0.22	1.75 ± 0.35	2.91 ± 0.22^∗^	3.53 ± 0.21^∗^
*Pear-shaped erythrocytes (%)*				
30	2.90 ± 0.48	2.92 ± 0.32	2.95 ± 0.29	2.99 ± 0.25
60	2.84 ± 0.62	2.95 ± 0.28	3.90 ± 0.21^∗^	4.51 ± 0.24^∗^
90	2.89 ± 0.65	2.97 ± 0.35	4.28 ± 0.22^∗^	5.12 ± 0.23^∗^
*Erythrocytes with micronucleus (%)*				
30	1.75 ± 0.34	1.77 ± 0.17	1.80 ± 0.22	1.82 ± 0.25
60	1.76 ± 0.38	1.79 ± 0.17	2.68 ± 0.21^∗^	3.09 ± 0.28^∗^
90	1.74 ± 0.41	1.82 ± 0.21	3.16 ± 0.17^∗^	3.78 ± 0.25^∗^
*Erythrocytes with condensed nucleus (%)*				
30	2.13 ± 1.12	2.15 ± 0.26	2.18 ± 0.25	2.21 ± 0.56
60	2.09 ± 0.61	2.16 ± 0.22	3.08 ± 0.31^∗^	3.54 ± 0.32^∗^
90	2.16 ± 0.82	2.21 ± 0.25	3.56 ± 0.18^∗^	4.17 ± 0.29^∗^
*Spindle-shaped erythrocytes (%)*				
30	1.42 ± 0.21	1.46 ± 0.22	1.47 ± 0.25	1.50 ± 0.27
60	1.47 ± 0.26	1.48 ± 0.33	2.37 ± 0.021^∗^	2.94 ± 0.025^∗^
90	1.46 ± 0.22	1.51 ± 0.26	2.81 ± 0.26^∗^	3.64 ± 0.34^∗^
*Spherocytes (%)*				
30	1.93 ± 0.25	1.95 ± 0.21	1.97 ± 0.26	1.98 ± 0.17
60	1.94 ± 0.13	1.96 ± 0.29	2.91 ± 0.25^∗^	3.49 ± 0.25^∗^
90	1.96 ± 0.26	1.99 ± 0.28	3.41 ± 0.22^∗^	4.18 ± 0.26^∗^

Values (mean ± SE) containing asterisks (^∗^) in each row differ significantly (*p* < 0.05) different to normal group.

**Table 3:** GSH contents, total proteins, and quantity of antioxidant enzymes in the liver of fish exposed to different salinities of brackish water.

Parameters/days	Groups (different salinities of brackish water)			
	T0 (0.0 ppt)	T1 (3.0 ppt)	T2 (5.0 ppt)	T3 (7.0 ppt)
*Liver*				
Reactive oxygen species (ROS) contents (optical density)				
30	0.13 ± 0.03	0.14 ± 0.02	0.15 ± 0.01	0.16 ± 0.04
60	0.14 ± 0.04	0.15 ± 0.04	0.21 ± 0.03^∗^	0.23 ± 0.03^∗^
90	0.15 ± 0.02	0.16 ± 0.01	0.24 ± 0.02^∗^	0.27 ± 0.02^∗^
*Thiobarbituric acid reactive substances (TBARS) contents (nmol/TBARS formed/mg protein/min)*				
30	24.3 ± 0.42	25.6 ± 0.40	25.8 ± 0.60	26.4 ± 0.23
60	24.5 ± 0.45	25.9 ± 0.25	27.2 ± 0.26	29.6 ± 0.51^∗^
90	24.4 ± 0.10	26.3 ± 0.28	30.7 ± 0.73^∗^	31.8 ± 0.47^∗^
*Reduced glutathione GSH (μmol/g tissue)*				
30	5.55 ± 0.02	5.48 ± 0.01	5.44 ± 0.01	5.36 ± 0.02
60	5.58 ± 0.01	5.43 ± 0.09	5.36 ± 0.01	4.01 ± 0.01^∗^
90	5.61 ± 0.25	5.31 ± 0.14	4.05 ± 0.03^∗^	3.92 ± 0.04^∗^
*Total proteins (μg/mg tissue)*				
30	6.29 ± 0.02	6.21 ± 0.01	6.11 ± 0.01	6.04 ± .03
60	6.30 ± 0.01	6.14 ± 0.03	5.96 ± 0.03	4.59 ± 0.01^∗^
90	6.33 ± 0.03	6.04 ± 0.02	4.55 ± 0.04^∗^	4.48 ± 0.27^∗^
*Superoxide dismutase SOD (units/mg protein)*				
30	11.3 ± 0.05	11.1 ± 0.06	10.8 ± 0.03	10.7 ± 0.05
60	11.5 ± 0.04	10.8 ± 0.03	8.73 ± 0.05^∗^	8.61 ± 0.02^∗^
90	11.6 ± 0.02	10.3 ± 0.02	7.8 ± 0.10^∗^	7.1 ± 0.23^∗^
*Catalase CAT (units/min)*				
30	6.46 ± 0.03	6.43 ± 0.01	6.40 ± 0.02	6.38 ± 0.03
60	6.43 ± 0.02	6.39 ± 0.01	4.81 ± 0.01^∗^	4.78 ± 0.02^∗^
90	6.40 ± 0.02	6.35 ± 0.02	4.68 ± 0.05^∗^	4.46 ± 0.01^∗^
*Peroxidase POD (units/min)*				
30	4.62 ± 0.05	4.48 ± 0.04	4.44 ± 0.05	4.37 ± 0.01
60	4.64 ± .05	4.42 ± 0.03	3.40 ± 0.03^∗^	3.31 ± .01^∗^
90	4.60 ± .01	4.38 ± 0.01	3.27 ± 0.01^∗^	3.17 ± 0.05^∗^

Values (mean ± SE) containing asterisks (^∗^) in each row differ significantly (*p* < 0.05) different to normal group.

**Table 4:** Oxidative stress parameters, GSH contents, and total proteins along with quantity of various antioxidant enzymes in the kidneys of fish exposed to different salinities of brackish water.

Parameters/days	Groups (different salinities of brackish water)			
	T0 (0.0 ppt)	T1 (3.0 ppt)	T2 (5.0 ppt)	T3 (7.0 ppt)
*Kidney*				
Reactive oxygen species (ROS) contents (optical density)				
30	0.28 ± 0.02	0.30 ± 0.02	0.31 ± 0.03	0.33 ± 0.01
60	0.29 ± 0.01	0.32 ± 0.01	0.33 ± 0.01	0.39 ± 0.04^∗^
90	0.31 ± 0.04	0.34 ± 0.03	0.38 ± 0.02^∗^	0.42 ± 0.03^∗^
*Thiobarbituric reactive substances (TBARS) contents (nmol/TBARS formed/mg protein/min)*				
30	32.6 ± 0.29	33.7 ± 0.60	34.2 ± 0.85	35.7 ± 0.42
60	32.9 ± 0.63	34.1 ± 0.24	35.8 ± 0.32	37.4 ± 0.21^∗^
90	33.5 ± 0.29	34.8 ± 0.43	36.5 ± 0.35	38.4 ± 0.27^∗^
*Reduced glutathione GSH (μmol/g tissue)*				
30	4.33 ± 0.06	4.27 ± 0.05	4.24 ± 0.03	4.20 ± 0.01
60	4.32 ± 0.01	4.20 ± 0.03	4.17 ± 0.04	4.11 ± 0.03
90	4.38 ± 0.03	4.18 ± 0.02	3.72 ± 0.06^∗^	3.65 ± 0.02^∗^
*Total proteins (μg/mg tissue)*				
30	5.63 ± 0.03	5.57 ± 0.05	5.51 ± 0.02	5.46 ± 0.04
60	5.65 ± 0.25	5.50 ± 0.03	5.43 ± 0.01	5.35 ± 0.07
90	5.66 ± .01	5.44 ± 0.02	3.98 ± 0.03^∗^	3.62 ± 0.02^∗^
*Superoxide dismutase SOD (units/mg proteins)*				
30	12.4 ± 0.25	12.0 ± 0.16	11.7 ± 0.17	11.3 ± 0.02
60	12.6 ± 0.06	11.9 ± 0.03	11.5 ± 0.11	10.13 ± 0.05^∗^
90	12.9 ± 0.13	11.6 ± 0.03	10.17 ± 0.02^∗^	10.01 ± 0.04^∗^
*Catalase CAT (units/min)*				
30	4.18 ± 0.04	4.02 ± 0.02	3.94 ± 0.04	3.87 ± 0.01
60	4.16 ± 0.01	3.97 ± 0.01	3.88 ± 0.04	3.76 ± 0.02
90	4.13 ± 0.03	3.88 ± 0.03	3.22 ± 0.03^∗^	3.11 ± 0.01^∗^
*Peroxidase POD (units/min)*				
30	3.87 ± 0.03	3.79 ± 0.01	3.70 ± 0.02	3.63 ± 0.05
60	3.83 ± 0.02	3.74 ± 0.01	3.61 ± 0.02	3.42 ± 0.01
90	3.76 ± 0.23	3.67 ± 0.02	2.36 ± 0.02^∗^	2.18 ± 0.03^∗^

Values (mean ± SE) containing asterisks (^∗^) in each row differ significantly (*p* < 0.05) different to normal group.

**Table 5:** Oxidative stress parameters, GSH contents, and total proteins along with quantity of various antioxidant enzymes in the gills of mrigal fish exposed to different salinities of brackish water.

Parameters/days	Groups (different salinities of brackish water)			
	T0 (0.0 ppt)	T1 (3.0 ppt)	T2 (5.0 ppt)	T3 (7.0 ppt)
*Gills*				
Reactive oxygen species (ROS) contents (optical density)				
30	0.15 ± 0.02	0.16 ± 0.03	0.18 ± 0.04	0.19 ± 0.03
60	0.16 ± 0.04	0.17 ± 0.01	0.22 ± 0.03^∗^	0.26 ± 0.01^∗^
90	0.16 ± 0.01	0.19 ± 0.04	0.23 ± 0.05^∗^	0.29 ± 0.04^∗^
*Thiobarbituric acid reactive substances (TBARS) contents (nmol/TBARS formed/mg protein/mg)*				
30	34.0 ± 0.47	35.4 ± 0.45	36.9 ± 0.39	37.2 ± 0.23
60	34.3 ± 0.23	36.1 ± 0.29	39.3 ± 0.21	41.5 ± 0.20^∗^
90	34.5 ± 0.28	37.2 ± 0.40	41.8 ± 0.59^∗^	43.3 ± 0.37^∗^
*Reduced glutathione GSH (μmol/g tissue)*				
30	1.36 ± 0.05	1.23 ± 0.02	1.21 ± 0.04	1.17 ± 0.02
60	1.37 ± 0.03	1.21 ± 0.01	1.04 ± 0.06^∗^	1.03 ± 0.03^∗^
90	1.37 ± 0.04	1.14 ± 0.03	1.01 ± 0.03^∗^	0.96 ± 0.01^∗^
*Total proteins (μg/mg tissue)*				
30	5.39 ± 0.03	5.34 ± 0.10	5.29 ± 0.04	5.18 ± 0.26
60	5.37 ± 0.14	5.30 ± 0.08	4.14 ± 0.02	3.81 ± 0.04^∗^
90	5.35 ± 0.02	5.27 ± 0.02	3.87 ± 0.03^∗^	3.78 ± 0.12^∗^
*Superoxide dismutase SOD (units/mg proteins)*				
30	8.65 ± 0.04	8.53 ± 0.01	8.44 ± 0.01	8.32 ± 0.01
60	8.64 ± 0.04	8.49 ± 0.03	6.41 ± 0.02^∗^	6.25 ± 0.02^∗^
90	8.64 ± 0.09	8.42 ± 0.05	6.15 ± 0.04^∗^	6.11 ± 0.05^∗^
*Catalase CAT (units/min)*				
30	2.81 ± 0.02	2.72 ± 0.03	2.64 ± 0.01	2.62 ± 0.02
60	2.85 ± 0.03	2.69 ± 0.02	2.17 ± 0.02^∗^	2.07 ± 0.01^∗^
90	2.84 ± 0.03	2.61 ± 0.05	2.15 ± 0.25^∗^	2.04 ± 0.04^∗^
*Peroxidase POD (units/min)*				
30	0.31 ± 0.01	0.29 ± 0.01	0.27 ± 0.01	0.26 ± 0.02
60	0.32 ± 0.03	0.28 ± 0.02	0.26 ± 0.03^∗^	0.25 ± 0.04^∗^
90	0.33 ± 0.05	0.27 ± 0.04	0.23 ± 0.06^∗^	0.21 ± 0.07^∗^

Values (mean ± SE) containing asterisks (^∗^) in each row differ significantly (*p* < 0.05) different to normal group.

## Data Availability

All the data related to the study is mentioned in the manuscript.
